# Supplementary material for: The relationship between the neutrophil percentage to albumin ratio and the occurrence of prostate cancer
Source: PLoS One. 2025 Nov 4;20(11):e0334426. doi: 10.1371/journal.pone.0334426 (PMC12585082; doi:10.1371/journal.pone.0334426)
Supplement: Attachment — Submitted filename: Response to Reviewers.docx [file pone.0334426.s002.docx]

Journal:**PLOS ONE**

Manuscript ID:PONE-D-25-16815

Dear editor and reviewer(s):

Thank you for email dated on September 03, 2025, about our manuscript entitled “The relationship between the neutrophil percentage to albumin ratio and the occurrence of prostate cancer”. We sincerely appreciate your valuable comments and suggestions and have revised our manuscript accordingly, providing a point-by-point response to each of them. And we have noted in details. To facilitate your review, the serial number preceding each comment corresponds to the numbered annotation in the revised manuscript.

**To respected editor:**

1.Limitations of Cross-Sectional Design and Self-Reported Data: Thank you for raising such a well-articulated points. We have stressed these limitations and their implications both in the Abstract and the Discussion section.

2.Causality: Language implying causality should be carefully revised. Thank you for pointing out this shortcoming. Revisions have been made in the manuscript.

3.Misclassification Bias: Self-reported cancer diagnoses are prone to significant misclassification bias. We have comprehensively addressed the potential implications of this issue on the results in the Discussion section. We acknowledge that the lack of sensitivity analysis for this potential bias is a limitation of our study. We appreciate your comment. This point will be thoroughly addressed in the Discussion section, and further investigation in future research is warranted.

4.Insufficient Detail on the Clinical Validation Cohort: We provided a table of baseline clinical characteristics, including age, BMI, smoking status, alcohol drinking, CVD, hypertension,diabetes and NPAR. All participants were of Han Chinese ethnicity. Variables such as Education and Poverty-to-income ratio were challenging to collect in the clinical setting due to issues including patients refusing to answer or being unable to recall the information. We acknowledge this limitation, which should be addressed in future studies.

5.Please provide more context on the clinical diagnoses. Thank you for bringing up this issue; it contributes significantly to the study's rigor. We have added a statement at the end of the "Assessment of prostate cancer" subsection in the Methods section.

6.The authors should clarify if PSA data were available and, if so, why they were not included in the model. If unavailable, this should be stated as a major limitation. Thank you for your thought-provoking question. We have acknowledged this limitation in the concluding part of the Discussion section. The detailed reasons are presented in the article.

7.Improper Citation: Reference [8] has been updated.

8.NEUT represents the neutrophil percentage (%), not the absolute count. We have revised in the Methods section.

9.Vague Language：We have revised the conclusion at the end of the Abstract section.

10.Figure Clarity：We have revised Figure 4.

Additional requirements:

The email of an institutional representative who is not involved in the study nor listed as an author of the manuscript, and who is capable of preserving the data and responding to external data access requests: **yudx_urology@163.com** The data have been deposited in Mendeley Data and will be retained long-term in the hospital's medical records archival system, guaranteeing persistent storage and ongoing accessibility.

Please update your Data Availability statement in the submission form accordingly: The Data Availability statement has been updated.

**To respected reviewer(s):**

11.PCa status was self-reported, introducing potential bias in classification. Please acknowledge this more directly in the limitations. We have already incorporated and emphasized this point in the limitations section.

12.The cross-sectional design prohibits inference of causality. Emphasize this more clearly in your discussion and conclusion. We have emphasized this more clearly in discussion and conclusion.

13.Important clinical factors such as PSA screening frequency and medication (e.g., statins, anti-inflammatories), and family history use are not accounted for. Note this as a limitation. This point has been incorporated into the limitations.

14.Clarify the thresholds used for categorizing NPAR and their applicability across diverse populations. We have refined both the Methods and Discussion sections.

15.Please reframe NPAR as a potential complementary marker rather than a standalone diagnostic marker. We have reframed it in Conclusion section.

16.Biological Plausibility: We have supplemented this point in the Discussion section.

Once again, we sincerely thank the editor and reviewer(s) for their positive and constructive feedback. We have incorporated all suggestions and are confident that the revisions have further strengthened the manuscript. We appreciate your consideration of our work for publication.

With best regards,

Sincerely yours,

Zhiqiang Zhang
